# Biofilm Bacterial Dynamics and Changes in Inorganic Nitrogen Density Due to the Presence of Freshwater Pearl Mussels

**DOI:** 10.1128/msphere.00834-21

**Published:** 2022-02-09

**Authors:** Kayano Takeuchi, Motoi Takeuchi, Wataru Kakino, Yutaka Uyeno

**Affiliations:** a Faculty of Agriculture, Shinshu University, Nagano, Japan; b Iwate Prefectural Kuji High School, Hirono, Iwate, Japan; c Division of Environmental Bioscience, School of Veterinary Medicine, Kitasato University, Towada, Aomori, Japan; University of Wisconsin—Madison

**Keywords:** biodeposit, freshwater bivalves, nitrogen balance, nutrient loading

## Abstract

The freshwater pearl mussel (genus *Margaritifera*) has shown severe declines, while the mussels play important roles in the translocation of nutrients and materials in river water ecosystems. We hypothesized that the biofilm bacterial composition and nutrient flow may reflect the differences in the existence of mussels. We analyzed water from 14 rivers from in multiple regions of Japan, including eight rivers, where the two species of freshwater pearl mussels (*Margaritifera laevis* and *Margaritifera togakushiensis*) are predominantly found, to analyze the microbial and nutritional nature of the biofilm artificially formed in the river. Field-produced biofilms, including the bacterial community structure, were examined, using next-generation sequencing of bacterial 16S rRNA gene amplicons followed by analyzing the genomic DNA extracted from the samples, inorganic nitrogen compounds, and chlorophyll *a* concentration. Compared to those in the control river without freshwater pearl mussels, biofilms of the existing river contained less inorganic nitrogen (ammonia and nitrate), suggesting the involvement of mussels in regulating the river water nutrient flow. Distinct changes were found in biofilms, depending on mussel existence, particularly in biofilms containing fewer photosynthetic bacterial groups, such as *Betaproteobacteria* and *Cyanobacteria*. Conversely, bacteria belonging to *Bacteroidales* in *Bacteroidetes* and *Clostridiales* in *Firmicutes* were predominantly found in biofilm samples where the mussels existed. Mussels alleviated strict nitrogen limitation in streams and possibly caused a concomitant change in the bacterial communities, where populations of bacterial groups exchanging inorganic nitrogen were low. We demonstrate the profound influence of freshwater mussel species on ecosystem processes and community dynamics across rivers.

**IMPORTANCE** The abundance of freshwater unioid mussels exhibited more diverse patterns of inorganic nitrogen flow and bacterial communities than the areas without mussels. This study demonstrates the effect of mussels on different freshwater ecosystem processes with variable organismal densities and biogeochemical factors. Freshwater unionid mussels significantly affect the ecosystem and community dynamics by modulating the relationships, altering nutrient availability, and indirectly manipulating the downstream ecological members, eventually expanding their role in the river ecosystems.

## INTRODUCTION

Freshwater mussels (*Bivalvia*: *Unionidae*) are large, long-lived (6 to 100 years) filter-feeding mollusks that occur in dense and large aggregations in river ecosystems. As many similar mussel assemblages have already been extirpated from rivers, freshwater pearl mussels (FPMs), which have been facing global decline and have been imperiled for the past 50 years, have attracted much attention from national and international conservation organizations (1–4). There are two species of margaritiferids (Margaritifera laevis and Margaritifera togakushinesis) in the family of *Margaritiferidae*, which are inhabitants of the cool and clean running waters of the Japanese archipelago in the southeast end of the Holarctic region (5, 6). Mussels depend on a well-oxygenated and stable substrate with high sedimentation quality and intense exchange between free-flowing and interstitial water (7). More importantly, the translocation and transformation of nutrients by FPMs are likely an influential biogeochemical process (8–11). In such nutrient cycling driven by these organisms, supporting a substantial proportion of nutrient demand (12), mussels enhance primary production and can lead to enrichment associated with community composition and function (13, 14). Accordingly, loss of the species would change the community composition and properties of riverine ecosystems and may drive the extinction of other species.

**FIG 1:**
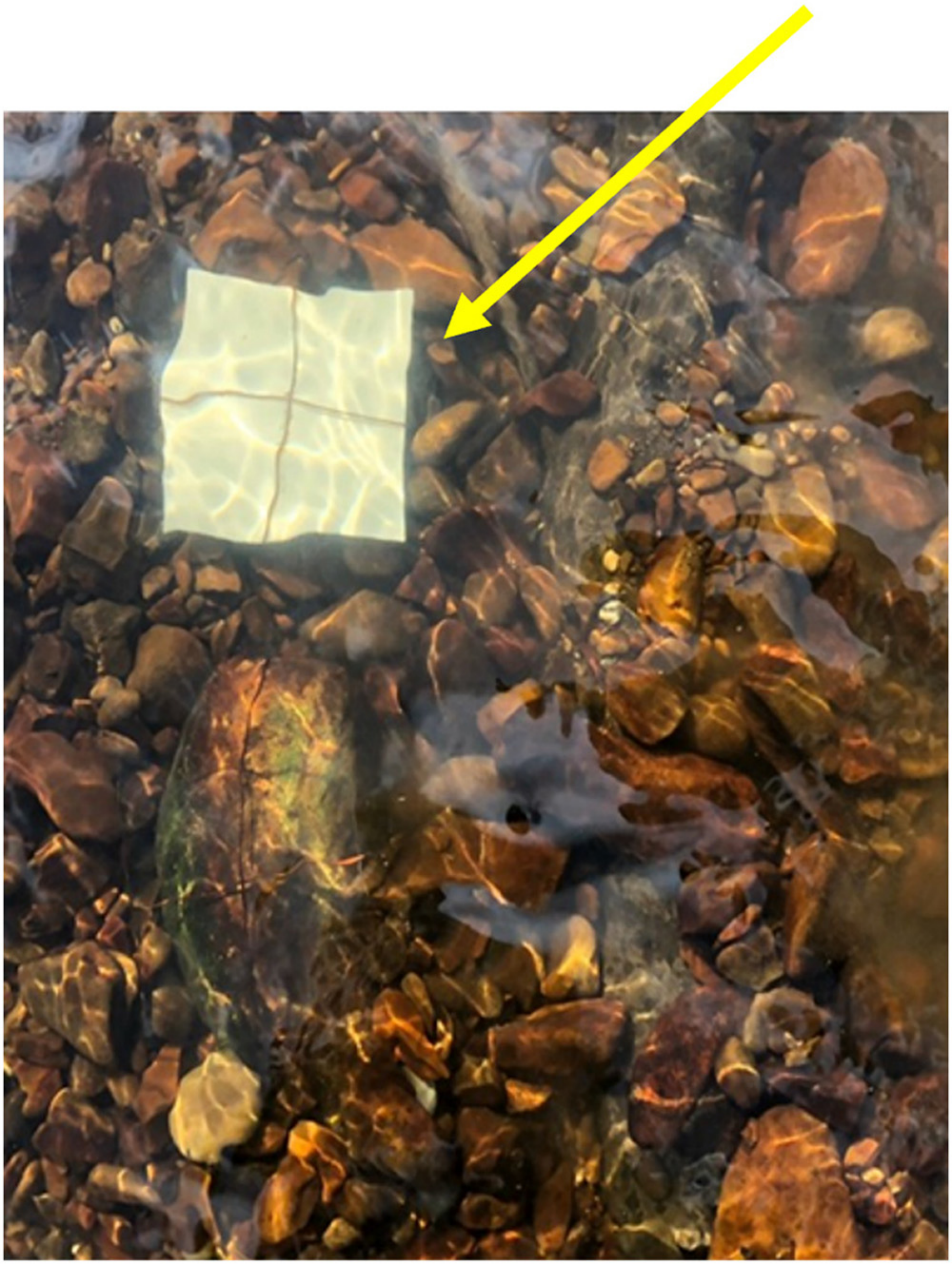
Representative image of the sampling tile (arrowhead in yellow) used for collecting biofilm from the river. The tile was tightly joined to a flat stone by using a thin wire to minimize the outflow.

Under the current preservation policy, it is difficult to directly investigate the organisms recognized for intensive action, and there is a dilemma regarding effective conservation. We alternatively focused on biofilms, the biomaterial closer to the mussels, which are easy to handle, and less disturbing for freshwater environments. Biofilms are involved in the complex life cycle of FPMs: for instance, the biofilm supports the metamorphosis of various species of oysters as a nutrient (food) in the post-secession from parasitizing fish and vice versa in an elder stage (15). The interaction of ecological systems and viable life determines the dynamics, local occurrence, or extinction of mussel populations. Moreover, differences in nutritional distribution in biofilm may determine the characteristics in nutritional flow of freshwater ecosystems. Although there are a few reports elucidating the intricate correlation between the inhabitation of these *Margaritifera* species and stream flow quality and physical environmental conditions, including the bottom sediment (16, 17), no substantial information is available about the effect of *Margaritifera* species on the microbial ecology in naturally occurring biofilms. Hence, further research is warranted to gain insights into the importance and magnitude of advantages of having mussels in an ecosystem. To address this, we designed this study to analyze the river water and the biofilm samples collected from ceramic tiles and examined the regional differences in the field-produced biofilms and the impact of FPMs on them. A sample of one of the ceramic tiles used is shown in Fig. 1.

## RESULTS AND DISCUSSION

**FIG 2:**
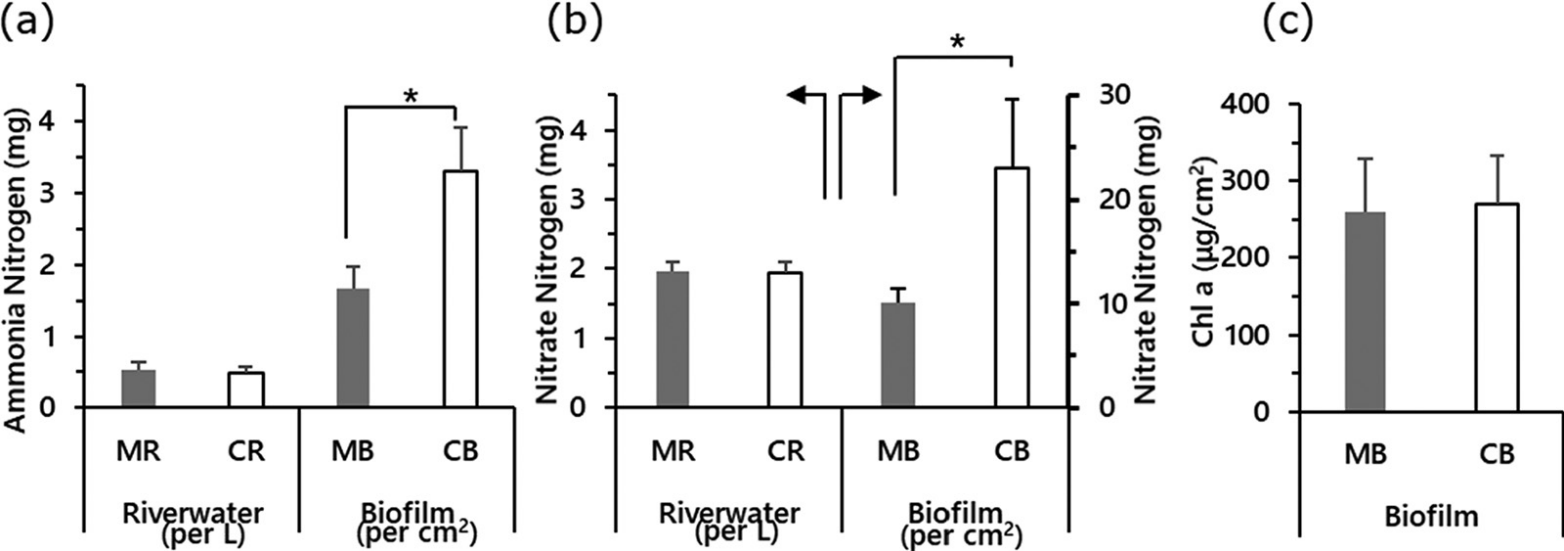
Chemical profiles of samples. (a) Ammonia nitrogen. (b) Nitrate nitrogen. (c) Chlorophyll *a*. MR, family *Margaritiferid*-positive river; CR, control (*Margaritiferid*-negative) river; MB, biofilm formed in MR; CB, biofilm formed in CR. Error bars indicate the standard error. *, *P* < 0.05.

**FIG 3:**
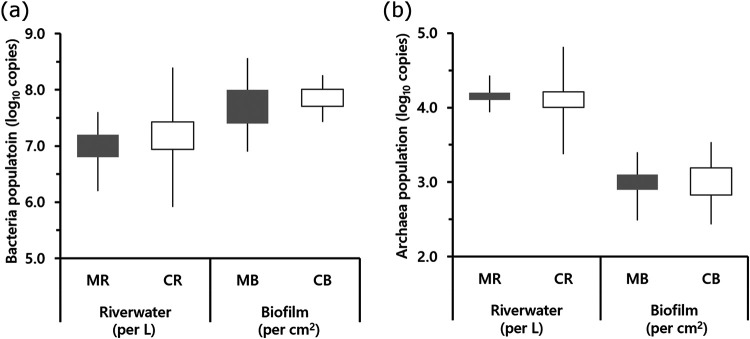
Bacterial and archaeal populations in the samples. (a) Log_10_-transformed bacterial 16S rRNA gene count and (b) log_10_-transformed archaeal 16S rRNA gene count. Data are shown in box plots, where the box represents the quartile interval and whiskers indicate minimum and maximum values. Abbreviations are the same as in Fig. 2.

**FIG 4:**
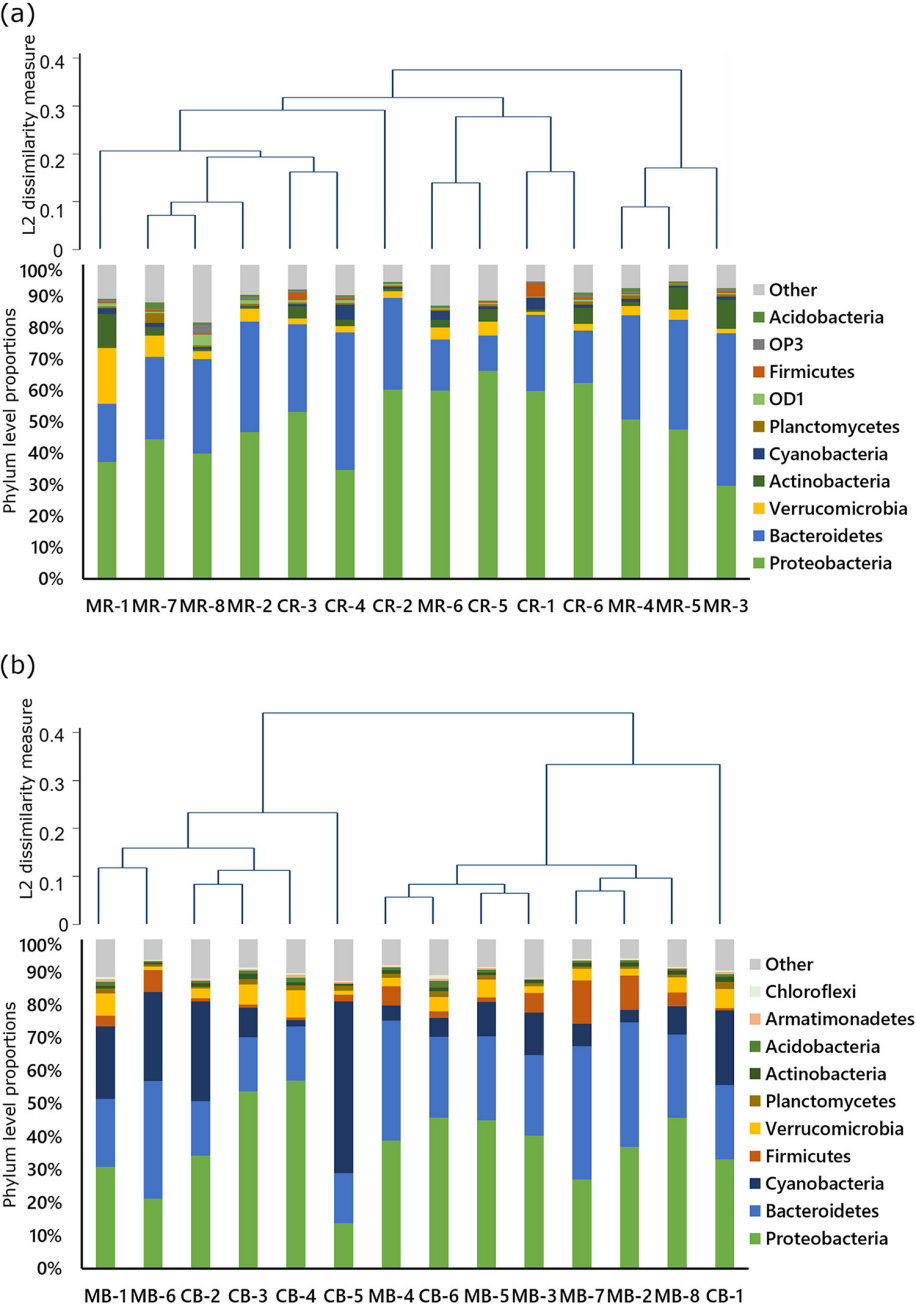
Phylogenetic distribution of bacteria in each sample. Samples were horizontally sorted according to the order in the L2 dissimilarity matrix tree, as described in the above distribution bars. The identity of each sample is explained in Table 1.

**FIG 5:**
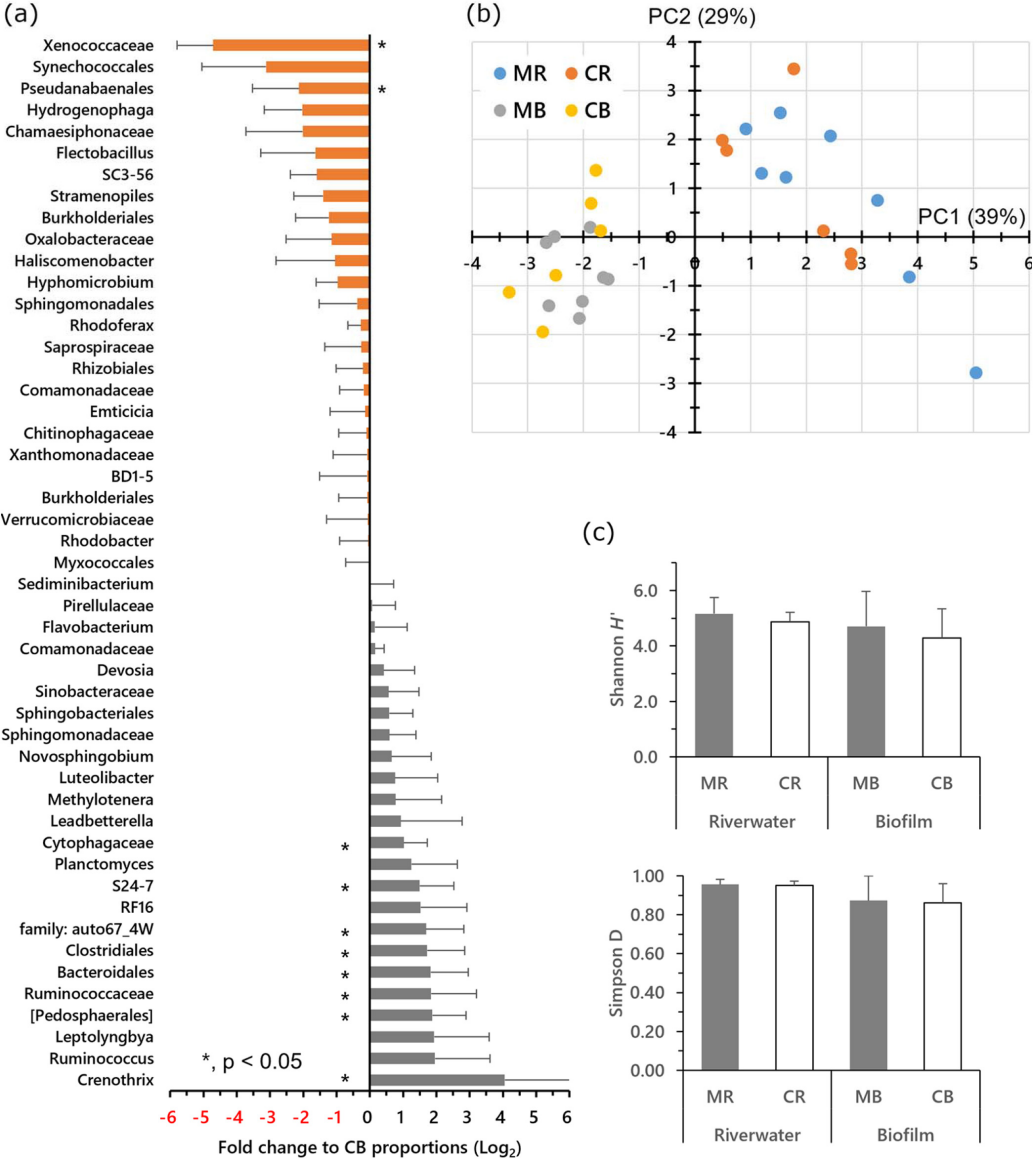
Transformed information about bacterial community structure among samples. (a) Fold change of MB to CB samples in the abundance of distinct phylogenetic groups at a taxonomic level lower than the family level. (b) Principal-coordinate analysis of samples at the operational taxonomic unit level using the weighted Unifrac distance metric, with samples labeled by sample attributions (MB, CB, MR, and CR) as indicated in the key. (c) Alpha diversity indices are indicated by two measurements: Shannon *H*′ and Simpson *D*. Abbreviations and error bars are the same as in Fig. 2.

Although there were no significant differences in inorganic nitrogen content between the MR (rivers with FPMs) and the CR (control rivers without FPMs) samples, significant differences were found in lower NH_3_-N and lower NO_3_-N of MB (biofilm formed in MR) than in CB (biofilm formed in CR) (Fig. 2a and b). Chlorophyll *a* contents were not significantly different among the biofilm samples (Fig. 2c). Between stream samples (MR and CR), no significant differences in total numbers of bacteria and archaea were observed (Fig. 3). In biofilm samples, the proportion of *Archaea* particularly was very small, and more bacteria were found than in stream samples. The bacterial community typically consisted of *Proteobacteria*, *Bacteroidetes*, and *Verrucomicrobia* in stream flow samples (Fig. 4a). Community similarity assessment based on the genus-level population highlighted that the structural differences were small in MB-2, -3, -4, -5, -7, and -8, as shown in Fig. 4b. The phylum *Proteobacteria* was the dominant group in all biofilm samples. A lower proportion of *Cyanobacteria* and higher proportions of *Betaproteobacteria* and *Gammaproteobacteria* were detected in MB than in CB. A lower proportion of *Cyanobacteria* was detected in the MB samples. On the other hand, particularly *Methylococcales* belonging to the *Gammaproteobacteria* and *Rhodoferax* in *Comamonadaceae* belonging to the *Betaproteobacteria* were also higher in MB than in CB (Fig. 5a). The results of the principal-component analysis (PCA) of MB and CB differed remarkably from those of freshwater samples. The PCA results revealed a smaller (shrinking) area of coverage in MB samples compared to that of CB samples (Fig. 5b). Cluster analysis showed that the communities in the biofilm samples differed from those in the stream flow samples. While the alpha diversity (expressed as in Shannon *H*′ and Simpson index) was not significantly different between MB and CB (Fig. 5c), the variation in bacterial community structure distinguished each from the other.

### Bacterial composition in freshwater and biofilm.

As shown in Fig. 4 and 5, the bacterial community in freshwater samples from different locations consisted of similar members. Prominent phyla were *Proteobacteria*, *Bacteroidetes*, and *Verrucomicrobia*, followed by *Actinobacteria*, *Cyanobacteria*, and *Planctomyces*. The proportional distribution of these phyla; however, was different. Detailed comparisons at the genus level exhibited compositional and proportional differences, in which the scattering of PCA data about freshwater samples reflected such diversity. There was a small deviation in alpha diversity parameters in freshwater samples, implying specific community structures among rivers in response to their respective ecological characteristics. Accordingly, there was no significant difference in the freshwater bacterial community between the two groups MR and CR. This may have reflected a consequent profile of different conditions among rivers—for example, water temperature, flow speed, and daylight intensity—reflecting the association of the structure of biofilm microbial communities with local environmental conditions and more prominent detection of *Cyanobacteria* (18, 19). On the other hand, significant differences in the bacterial proportions were found in *Bacteroidetes* and *Proteobacteria* between the MB and CB samples. A higher proportion of *Methylococcales* (belonging to the class *Betaproteobacteria*) may also imply the characteristics of biofilms in the presence of mussels. Bacteria belonging to this order have been recognized as methanotrophs (20, 21). Moreover, a very small number of methanogenic archaea were detected in the biofilm, regardless of the presence of FPMs. Therefore, the methanotrophs might depend on the methane molecules flowing from the sediments at the bottom of the river stream (22, 23). The results of the PCA of MB and CB remarkably differed from those of freshwater samples, indicating that the bacterial compositions in biofilms do not necessarily reflect those of freshwater but likely depend on the surrounding environment, which may involve the existence of other living members and molecules. For example, the Mizujinja waterway (CR-5) is a river that runs just beside the Hinokinai River branch (MR-6), where the FPMs live, and is thought to be the same water system that was also exhibited by the dendrogram ordering CR-5 next to MR-6 (Fig. 4a). Meanwhile, a concomitant interesting result was that the microbial proportions of MB-6 and CB-5 were considerably different. According to the community structure composition and PCA results, the microconsortium in MB samples may have comprised more consolidated members by presenting the effects of freshwater mussels through changes in nutrients, such as the proportions of nitrogenous compounds. Although MB-1 and -6 were separated from the MB cluster, the reason for this result may be relevant to the average age of mussels since there were larger mussels, as shown in Table 1. Longitudinal natural changes in river ecosystems may also account for some environmental and biological variations (24). Since there was no significant change in the alpha diversities between MB and CB, the degree of proportional distribution of bacteria was similar among samples, whereas the occupancies of the respective members were different.

**TABLE 1:** Sampling sites and conditions

Area	Mussel-positive river					Mussel-negative (control) river			
	Name (sample ID)^*a*^	Water temp (°C)	Depth (cm)	Width (m)	Shell length (cm)^*b*^	Name (sample ID)^*a*^	Water temp (°C)	Depth (cm)	Width (m)
Amori	Tanabu (MR-1 [<30/km^2^])	19.2	40∼60	20	11.9 (ML)	Biwano (CR-1 [980/km^2^])	11.6	28∼80	2.3∼3.4
	Chidorisawa (MR-2 [<30/km^2^])	16.8	7	1.7	6.6 (MT)	Imaizumi (CR-2 [<30/km^2^])	10.8	12∼30	2.0
Iwate	Kawajiri (MR-3 700/km^2^)	19.5	15∼23	14	9.7 (ML)	Toya (CR-3 [100/km^2^])	20.1	12	5.0
	Myonai (MR-4 [300/km^2^])	18.2	13∼21	0.7∼0.8	8.0 (ML)	Ube (CR-4 [240/km^2^])	16∼17	11	3.3
	Echizenzeki (MR-5 [400/km^2^])	15.7	30∼60	4.2∼6	7.7 (ML)				
Akita	Hinokinai (MR-6 [100/km^2^])	22.7	35	2.2	11.4 (ML)	Mizujinja (CR-5 [100/km^2^])	22.6	60	3.2
Nagano	Nougu (MR-7 [<30/km^2^])	20.3	5	1.9	9.3 (ML)	Inaosawa (CR-6 [120/km^2^])	14.9	20	2.2
	Sakasagawa (MR-8 [<30/km^2^])	13.6	15	3.5	7.7 (MT)				

a Numbers in brackets next to the sample ID represent the mean approximate population density (per km^2^) around the watershed of the sampling site, determined by mesh-basis data presentation by a public map service (jSTAT MAP [https://www.e-stat.go.jp/gis]); the population was based on the national census in Japan, taken on (1 October 2015).

b The observed *Margaritifera* species are indicated as *M. larvae* (ML) and *M. togakusinesis* (MT).

We also identified the family *Comamonadaceae* as another example with the proportional difference between MB and CB. This genus, which belongs to the order *Burkolderiales*, was prominent in all freshwater samples analyzed. This purple bacterium is a group of anaerobic photosynthetic bacteria; their function in the river stream would be less expected, while in the biofilm, they would function with photosynthetic *Cyanobacteria*, prospering as a major photosynthetic organism in freshwater systems at the same time as a dominant species during biofilm formation in rivers (24, 25). In MB, other anaerobic autotrophic bacteria of the genus *Rhodoferax* were rather prominent, suggesting that this genus contributed to the nutrient supply in the biofilm. On the other hand, MB samples were particularly enriched in *Firmicutes* and in the order *Bacteroidales*: both are typically heterotopic, as these are dominant in the animal intestine. This finding could be an important clue for understanding the essential contribution of mussels to river water nutrient circulation, as discussed later.

### Nutrient loading by FPMs through differential bacterial distribution in biofilms.

This report primarily aimed to elucidate the effects of adult freshwater mussels on nutrient flow in biofilms and, probably also, in river water ecosystems. In the biofilm, a significant difference was found in ammonia, and nitrate was higher in CB than in MB. In rivers and freshwater ecosystems, nutrient recycling is usually poor; therefore, adult mussels often aggregate into mussel beds, and they are patchily distributed in streams to play an important role in the translocation, storage, and recycling of nutrients (13, 26, 27). This performance by FPMs helps other members living in freshwater and indicates the co-occurrence of particular species adapted to cool oxygen-saturated running waters that carry a small amount of nutrients (28). In particular, adult freshwater mussels strongly affect coupled nitrification-denitrification by biodepositing organic material, moving it back into the stream in an inorganic form according to environmental demands, and mixing sediments as they move downstream (29, 30). Mussels are indispensable, since mussel excretion accounts for 40% of the nitrogen in a nutrient-limited river (13, 31).

Our results demonstrated that FPMs are involved in the changes in the biofilm bacterial composition and nutrient balance. Concerning inorganic nitrogen balance, a previous study described the bacterial community shift in sediment samples in response to NH_3_ release by FPMs with an increase in ammonia-oxidizing bacteria and nitrogen-transforming microorganisms (32). In the present study, some bacterial groups related to ammonia oxidization were detected, but the population was very low. For example, less than 0.01% of *Nitrosomonas* isolates in *Betaproteobacteria* were present in both CB and MB. With the reduction in inorganic nitrogen in the biofilm affected by the presence of FPMs, the nutrient contribution by FPMs to nearby ecological niches was preferable to biofilm community members to enable easier nitrogen utilization in the niche, instead of N-fixing algae (i.e., blue-green algae and *Epithemia*) (31). *Epithemia* drives cyanobacterial endosymbionts, which fix the atmospheric nitrogen, and may have occurred in CB but not in MB. Although mussels have been found in relatively eutrophic rivers, high ammonia concentrations are harmful to *Margaritifera*, and a close relationship between an increase in the nitrate concentration and a decrease in the mussel population has also been suggested (33). In our results, for example, average ammonia nitrogen concentrations were 1.33 μg/cm^2^ at Hinokinai (MR-6), while they were 4.73 μg/cm^2^ at Mizujinja (CR-5), implying that FPMs may have provided suitable nutrients in the form of feces to the biofilm bacterial community, which were very well utilized to build the film in less inorganic wastes, which provides preferable conditions for FPM survival. Since it has been recognized that the presence of FPMs is associated with photosynthetic organisms in biofilm structure and their nutritional balance, differences in the energy metabolism of photosynthetic organisms have been speculated in the presence of FPMs. In this study, instead of evaluating photosynthetic algae, we employed chlorophyll *a* as a quantitative indicator of algae and other photosynthetic organisms in freshwater. Our result was partly consistent with a previous report showing a reduction in *Cyanobacteria* in the river water with FPMs compared to the river water lacking FPMs, because of the direct consumption of *Cyanobacteria* by FPMs (34). Akiyama et al. analyzed FPM feces and indicated traces of algae as feed (17).In addition, we expected a reduction in chlorophyll *a* in rivers where FPMs were present, but no significant change was observed between MB and CB. This decrease may also explain why adult mussels affected the stream nutrient flow without consuming *Cyanobacteria* in these areas. Biofilms are known to facilitate the setting and metamorphosis of oyster larvae (35). Adult FPMs are likely to consume a large amount of algae in the biofilm as an important nutrient at the early stage, which may fulfill their nutritional requirements. Mussels deposit particulate materials into interstitial sediment as their feces or pseudofeces to provide soluble nutrients readily taken up by algae and heterotrophic bacteria (13, 27) and cascade up aquatic food webs. In relation to this, we speculate that our results would also be related to the preservation of juvenile mussels, one of the main concerns of reproduction in most existing FPM areas. Juvenile mussels are known to be more sensitive to ammonium than other freshwater organisms (36). Substrate factors probably also closely correlate with the productivity and food availability of young pearl mussels, a field that is still poorly investigated and understood. By tracing back lower nitrogen components of biofilm to an upstream place, even the presence of juvenile mussels can be determined, as host fish carry the glochidia upstream, where there is less inorganic nitrogen. To understand the influence of nutrient conditions on the animal nutrient dynamics and determination of the presence of juvenile mussels, more importance must be given to the preservation strategy.

**FIG 6:**
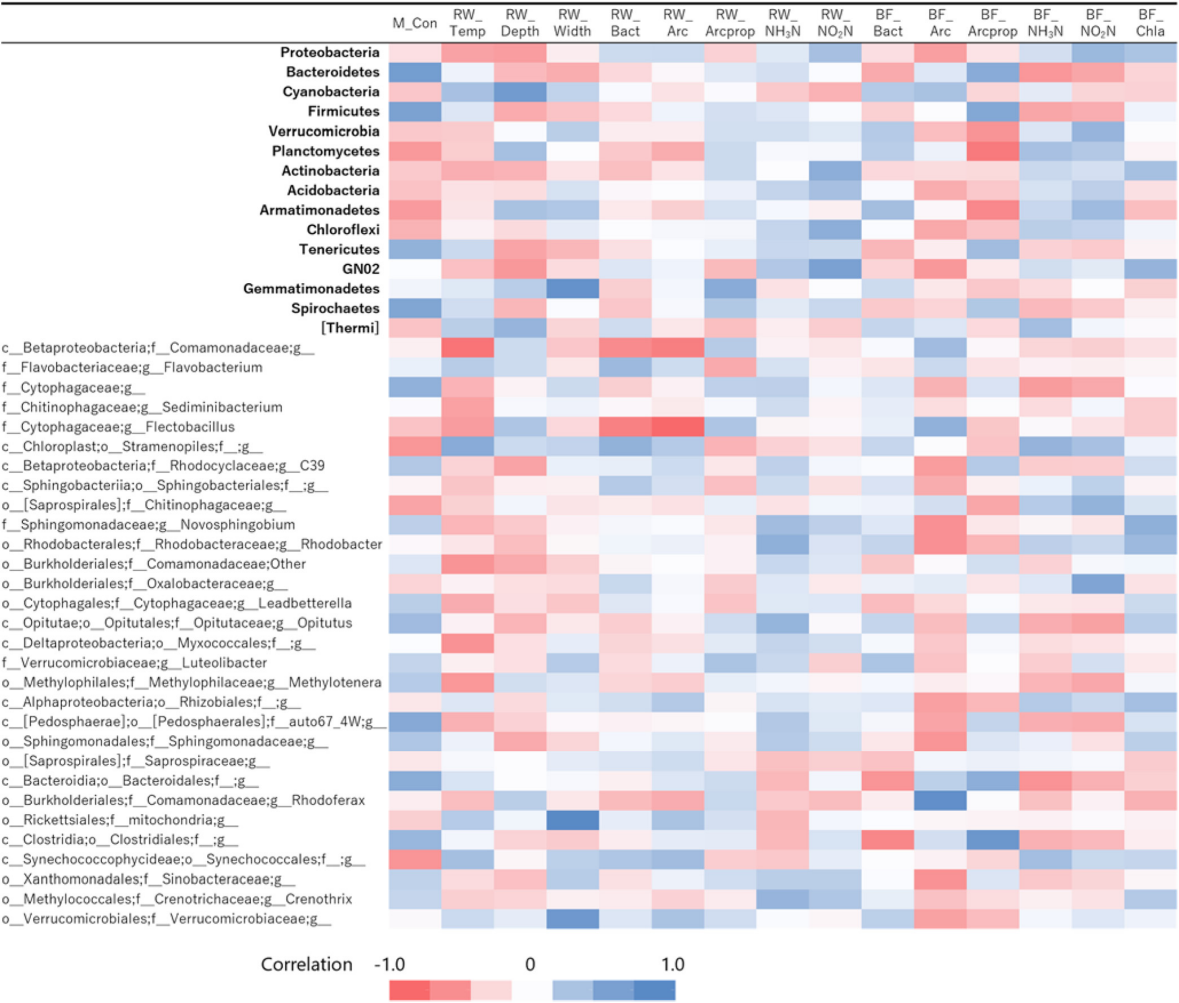
Correlation mapping of taxonomic distribution in biofilm samples and parameters in river waters and the biofilm samples. Matrix cell color represents positive (blue) or negative (red) correlations. M_Con, contrast of MB and CB, where dummy variables are assigned to MB = 1 and CB = 0; RW_Temp, river water temperature; RW_Depth, depth of the river; RW_Width, width of the river; RW_Bact, bacteria numbers of river water sample; RW_Arc, archeal numbers of the river water sample; RW_Arcprop, archeal proportion to bacteria of the river water sample; RW_NH_3_N, ammonia nitrogen in the river water sample; RW_NO_2_N, nitrate-nitrogen in the river water sample; BF_Bact, bacterial numbers of the biofilm sample; BF_Arc, archaeal numbers of the biofilm sample; BF_Arcprop, archeal proportion to bacteria of the biofilm sample; BF_NH_3_N, ammonia nitrogen in the biofilm sample; BF_NO_2_N, nitrate nitrogen in the biofilm sample; BF_Chla, chlorophyll *a* in the biofilm sample.

We further compared the data of the population of representative bacterial groups found in biofilm (regardless of the presence of FPMs) and biotic and abiotic parameters to generate a correlation matrix to depict which bacterial group in biofilm was presumably responsible for the surrounding properties (Fig. 6). This approach provided remarkable findings. A negative correlation between NH_3_-N in biofilm and bacterial groups was frequently detected in animal intestines (i.e., *Bacteroidales* in *Bacteroidetes*, *Firmicutes*, and *Tenericutes*). This relationship can be explained by the fact that ruminant animals rely on the protein synthesis capability of the bacterial members present in the forestomach. As such, the presence of bacterial groups frequently found in the animal gut may indirectly explain the important role of FPMs in biofilm formation by providing its intestinal matter as a nutrient.

### Conclusion.

The areas with mussel densities showed different patterns of inorganic nitrogen flow and bacterial communities compared to areas with no mussels. We have demonstrated the influence of mussels on freshwater ecosystem processes across locations where the organismal densities and biogeochemical factors varied. Freshwater unionid mussels profoundly impact the ecosystem and community dynamics by stipulating relationships by altering nutrient availability and indirectly controlling the downstream ecological members, to improve their role in the river ecosystems. We still have a poor understanding of the role and importance of these biodeposits in nutrient dynamics and food web support, and this requires more intensive research. In addition, more intensive ecological investigations targeting the nature of the habitat for freshwater pearl mussels and links between the pearl mussels, their ecosystems, and their importance for global biodiversity will be required.

## MATERIALS AND METHODS

### Study area and sampling.

We studied four prefectures in Honshu Island, Japan, where the FPM genus *Margaritifera* has been reported to survive (Table 1). We selected 14 sites for this study: eight sites with dense mussel aggregations in one bed and six sites without mussels. Following the national and regional regulations, we obtained permission for the temporary pickup of the mussels that have been listed within the Red List by Ministry of the Environment, Japan, and registered as natural monuments by regional authorities, and we surveyed nearby environmental parameters. We chose sites based on previous visual surveys conducted. Mussels were measured for length, width, and depth of each shell and were immediately placed back into the original habitat. All sites were located upstream of respective rivers, and mussel and no-mussel sites were similar in size and water chemistry (Table 1). The temperature of the stream flow was measured on-site. Information regarding the human population based on the data from the recent national census was also included in the table. Neither the river with mussels nor the control river had any contamination from human activities. The waters from both the sources were considered unpolluted based on the visual observation and presence of char (belonging to the genus *Salvelinus*), cherry trout (Oncorhynchus masou), Japanese fluvial sculpin (Cottus pollux), and aquatic plants that strictly require clear water for their growth. Mussel beds were diverse. Mussels made colonial aggregation typically with over 20 mussels per square meter, and beds were often separated by small distances from streams (1 to 5 m). We used a ceramic tile (10 cm by 10 cm) that was settled at a place close to the mussel beds and ambient river flow (positive site) or a place where there is less possibility of human intervention and a similar river flow (negative site). The tile was tightly joined to a flat stone using a thin wire (a representative image is provided in Fig. 1). After 2 weeks, we collected the tiles and 2 L of stream flow from rivers where FPMs existed (MR) or were absent (control river [CR]). Samples were kept at 4°C (stream flow) and frozen (tiles on which biofilm formed) until further analysis.

### Microbial analysis.

The total bacterial count and the number of methanogenic archaea from the 28 samples (14 stream flow samples and 14 biofilms [referred to as MB and CB]) were determined using a real-time PCR method. One liter of river water sample was filtered by vacuum aspiration using a cellulose ester membrane filter (Advantec A045A047A; Toyo Roshi, Ltd., Tokyo, Japan) and was subsequently used for gene extraction. The biofilm sample was uniformly scraped from different parts of the tile amounting up to half of its area (50 cm^2^). The obtained sample was weighed and used for experimentation, as described later. The genomic DNA of the microorganisms present in the samples was extracted using the Qiagen Powerwater DNA extraction kit (Qiagen, Hilden, Germany) according to the manufacturer’s protocol and stored below −20°C for further analysis. The PCR conditions and primer sequences for counting total bacteria used in this study were in accordance with those used in a previous study (37). Primer sets Eub338F (5′-ACTCCTACGGGAGGCAG-3′) and Eub522R (5′-ACGTCRTCCMCNCCTTCCTC-3′) for total bacteria, and the qmcrA primer set (5′-TTCGGTGGATCDCARAGRGC-3′ for the forward primer and 5′-GBARGTCGWAWCCGTAGAATCC-3′ for the reverse primer) for methanogenic *Archaea* (38), as well as a CFX96 real-time PCR detection system (Bio-Rad, Inc., Hercules, CA), and a SYBR Premix *Ex Taq* kit (TaKaRa Bio, Inc., Otsu, Japan), were used. The PCR cycling conditions were as follows: initial denaturation at 95°C for 10 s and 40 cycles at 95°C for 5 s and 62°C for 30 s. This was followed by melting curve analysis to confirm that the expected PCR products were obtained. Furthermore, the extracted bacterial genomic DNA was subjected to 16S rRNA gene amplicon pyrosequencing. Primer sets 515F (5′-GTGCCAGCMGCCGCGGTAA-3′) and 806R (5′-GGACTACHVHHHTWTCTAAT-3′), a T-100 thermal cycler (Bio-Rad, Inc., Hercules, CA), and an *Ex Taq* kit (TaKaRa Bio, Inc., Otsu, Japan) were used to generate the amplicons. The PCR cycling conditions used for amplification were initial denaturation at 95°C for 10 s and 25 cycles at 95°C for 10 s, 57°C for 30 s, and 62°C for 30 s for the first PCR, followed by 95°C for 10 s and 10 cycles at 95°C for 10 s, 57°C for 30 s, and 62°C for 30 s for the second PCR. A barcoded amplicon was subjected to paired-end sequencing on an Illumina MiSeq platform (Illumina, San Diego, CA, USA). Postanalyses of the sequencing results were conducted after filtering the low-quality reads and trimming the adapters, barcodes, and primers using QIIME. The reads from all samples were clustered into operational taxonomic units (OTUs) at a 97% sequence similarity level. The data for species-level taxonomy were obtained by filtering the OTU tables containing taxonomic data generated using the Ribosomal Database Project (RDP) Classifier at the genus level. Representative sequences were then extracted, and species-level matches within the National Center for Biotechnology Information database were identified using the Basic Local Alignment Search Tool (BLAST). Alpha diversity was measured using both the Shannon and Simpson indices.

### Chemical analysis.

The remaining biofilm on the tile was scraped and weighed prior to the analysis. The chlorophyll *a* content in biofilms was quantified using a spectrophotometer (18). Inorganic nitrogen (NO_3_-N) was determined using a quantification reagent kit for NH_3_-N (Labassay Ammonia, Fujifilm Wako Pure Chemical Co., Ltd., Osaka, Japan) and a direct measurement device (LAQUAtwin NO_3_-11; Horiba, Ltd., Kyoto, Japan) following the manufacturer’s protocols.

### Statistical analysis.

The analyzed data of the biofilms were expressed per area of the tile (i.e., per cm^2^). Since there is a possibility of obtaining biased information regarding biofilm formation on the tile, we compared the weights of two different subsamples for the bacterial DNA extraction, followed by chemical analysis, and reconfirmed that they genuinely were of the same weight. We used the following equation to normalize the data: corrected data (U/cm^2^) = [raw data (U)/factor]/area of the tile (100 cm^2^), where “factor” = weight of the subsample (g)/total biofilm weight on the tile (g).

Chemical parameters between the positive and negative sites were compared using Student’s *t* tests. The significance level was set at *P* < 0.05. To determine the beta diversity of the microbiota among samples, principal-component analysis (PCA) was applied to the bacterial composition of the OTU data sets of all (i.e., stream flow and biofilm) samples. All statistical treatments were performed using the STATA release 13.1 package (version 13.1; Stata Corp., College Station, TX, USA).

### Accession number(s).

The Illumina MiSeq platform sequencing results were submitted to the DNA Data Bank of Japan (DDBJ) Sequence Read Archive under accession numbers DRR320275 to DRR320302.
